# Our “Saving” Solons

**Published:** 1899-06

**Authors:** 


OUR "SAVING" SOLONS.—The one idea of the average Texas legislator seems to be "retrenchment,"—saving. The appropriation bill, after weeks of fighting, narrowing down sometimes to a wrangle over $2.50 per month on some poor devil of a clerk's salary, carrying the miserable pittance of $40,000 a year for the University of Texas and $35,000 for the Medical Department, was finally passed, after a tie vote in the House, by the vote of the Speaker. One old fellow is reported to have opposed university education "on general principles." He said "his daddy gave him his larnin,' and other feller's daddies could do the same for their boys."

---

WE EXTEND OUR CONDOLENCE to the brethren of Colorado. They had a bill to "regulate the practice," almost identical with our "Wilson bill," and after a desperate fight got it through both houses of the Legislature,—and then the Governor—"Thomas"—the Colorado journals call him, some adding "cat," vetoed it. The *Denver Medical Times* and the *Colorado Medical Journal* don't "do a *thing*" to him in the May issues. In Texas we couldn't get as far as the Governor; our bill was pigeon holed *early,* and one Davidson got up a better one (?) as he thought; but even that didn't make the trip.

---

REPEAL OF THE TAX ON DOCTORS.—The JOURNAL extends it congratulations to the physicians of Texas on their success with the Legislature in getting the obnoxious "occupation tax" law repealed. We ought to rejoice, as it is the only thing the doctors have ever been able to get them to do.

